# Co-existence of physiologically similar sulfate-reducing bacteria in a full-scale sulfidogenic bioreactor fed with a single organic electron donor

**DOI:** 10.1007/s00253-007-0968-y

**Published:** 2007-04-14

**Authors:** Shabir A. Dar, Alfons J. M. Stams, J. Gijs Kuenen, Gerard Muyzer

**Affiliations:** 1 Department of Biotechnology, Delft University of Technology, Delft, The Netherlands; 2 Laboratory of Microbiology, Wageningen University, Wageningen, The Netherlands

**Keywords:** Sulfate-reducing bacteria, Microbial ecology, Ecological niches

## Abstract

A combination of culture-dependent and independent methods was used to study the co-existence of different sulfate-reducing bacteria (SRB) in an upflow anaerobic sludge bed reactor treating sulfate-rich wastewater. The wastewater was fed with ethanol as an external electron donor. Twenty six strains of SRB were randomly picked and isolated from the highest serial dilution that showed growth (i.e. 10^8^). Repetitive enterobacterial palindromic polymerase chain reaction and whole cell protein profiling revealed a low genetic diversity, with only two genotypes among the 26 strains obtained in the pure culture. The low genetic diversity suggests the absence of micro-niches within the reactor, which might be due to a low spatial and temporal micro-heterogeneity. The total 16S rDNA sequencing of two representative strains L3 and L7 indicated a close relatedness to the genus *Desulfovibrio*. The two strains differed in as many as five physiological traits, which might allow them to occupy distinct niches and thus co-exist within the same habitat. Whole cell hybridisation with fluorescently labeled oligonucleotide probes was performed to characterise the SRB community in the reactor. The isolated strains *Desulfovibrio* L3 and *Desulfovibrio* L7 were the most dominant SRB, representing 30–35% and 25–35%, respectively, of the total SRB community. *Desulfobulbus*-like bacteria contributed for 20–25%, and the *Desulfobacca acetoxidans*-specific probe targeted approximately 15–20% of the total SRB. The whole cell hybridisation results thus revealed a consortium of four different species of SRB that can be enriched and maintained on a single energy source in a full-scale sulfidogenic reactor.

## Introduction

Many industrial processes, such as metal smelting, flue gas scrubbing and mining generate sulfate-rich wastewater (Lens et al. 1998). These wastewaters usually do not contain any organic carbon, and the addition of external electron donor is required for their treatment. These waste streams cause a range of problems associated with the now well-established anaerobic treatment of these wastewaters, such as a decrease in methane production (Colleran et al. 1995), sulfide toxicity (O’Flaherty and Colleran 2000), foul smell (Lens et al. 2001) and corrosion (Vincke et al. 2001). Of late, bioreactor processes have been developed for treating these sulfate and metal-rich wastewaters. This technology developed by the company Paques BV in The Netherlands (Pol et al. 2001) makes use of the dissimilatory sulfate-reducing capacity of sulfate-reducing bacteria (SRB) to simultaneously remove sulfate and metals in the form of metal sulfides. A typical application is a two-step process that includes dissimilatory sulfate reduction to sulfide (Gibson 1990) as the first step. Subsequently, the sulfide will be bound by heavy metals, such as cobalt, lead, nickel and zinc and precipitated as metal sulfide (Colleran et al. 1995). The second step is the biological oxidation of the remaining sulfide to insoluble elemental sulfur, which is either recovered by sedimentation or a small portion converted to H_2_SO_4_ and re-used to neutralise the alkalinity generated because of sulfide production in the first step.

Several full-scale sulfate removal bioreactors are currently in operation. The dominant sulfidogenic communities in these bioreactors are heterotrophic SRB belonging to the family *Desulfovibrionaceae* (Kaksonen et al. 2004; van Houten et al. 2006). The genus *Desulfovibrio* represents a phylogenetically coherent group; all species incompletely oxidise lactate to acetate but can utilise hydrogen, formate and ethanol as well (Widdel and Bak 1992).

In an engineered system fed with a single nutritional source and kept under constant operational parameters such as pH, temperature and salinity, competition among species might tend to reduce the species and sub-species diversity according to the principle of competitive exclusion (Gause 1934). However, physiologically competing species can co-exist if they occupy different niches, whereby each species uses distinct parts of the resource base. In the present study, we investigated the co-existence of physiologically similar hydrogenotrophic SRB in a full-scale sulfidogenic bioreactor treating sulfate-rich wastewater using a combination of cultivation and molecular techniques. The reactor was fed with ethanol as carbon and energy source. SRB isolated in pure culture were characterised genetically and physiologically. Micro-diversity among the dominant culturable isolates was assessed by repetitive enterobacterial palindromic polymerase chain reaction (rep-PCR) (Versalovic et al. 1994). Whole-cell hybridisation with fluorescently labelled general and specific probes was used for SRB community characterisation and for the estimation of the relative abundance of the different SRB populations in the reactor.

## Materials and methods

### Sludge source

Granular sludge was obtained from an upflow anaerobic sludge bed (UASB) reactor, treating sulfate-rich wastewater from a chemical plant located in Emmen, The Netherlands. The wastewater fed to the reactor did not contain any organic compound, so ethanol was added as an external electron donor and carbon source. The reactor had a volume of 300 m^3^ and a feed rate of 60–75 m^3^ h^−1^ The ratio between the amount of electron donor added and the amount of sulfate in the reactor was around 0.4 kg/kg. Sulfide produced in the reactor was either converted to elemental sulfur through biological sulfide oxidation or precipitated with toxic metals. The reactor was operated at a temperature of 30°C and a pH of 7.0–7.5. The concentration of sulfate in the wastewater was approximately 1,500 mg/l and ca. 100 mg/l in the effluent.

### Culture media and isolation of SRB

A basal bicarbonate-buffered and sulfide-reduced medium was used for the enumeration and isolation procedures. The mineral medium contained per litre of distilled water: 0.2 g KH_2_PO_4_, 0.25 g NH_4_Cl, 0.5 g KCl, 0.1 g CaCl_2_·2H_2_O, 0.4 g MgCl_2_·6H_2_O, 1.0 g NaCl and 0.5 ml of a resazurin solution (0.5 mg ml^−1^) as a redox indicator. The medium was supplemented with (per litre) the following: 30 ml 1 M NaHCO_3_ solution, 1 ml of a vitamin solution, 1 ml of trace element solution with ethylenediamine tetraacetic acid (EDTA; Widdel and Bak 1992) and 0.1 g of yeast extract. As a reducing agent, 7.5 ml l^−1^ of 0.2 M Na_2_S·9H_2_0 was added. Either lactate or ethanol (20 mM) was used as an electron donor and sulfate (10 mM) as electron acceptor. Enumeration of the potentially dominant heterotrophic sulfate reducers was performed by serial dilutions in Hungate tubes. The Hungate tubes were incubated at 30°C in the dark for 7 weeks. The highest positive dilution tubes that showed growth were selected for further isolation. Growth was assayed by measuring sulfide production photometrically (Cord-Ruwisch 1985).

Pure cultures were obtained by repeated transfer in agar shake tubes (Widdel and Pfennig 1984). Purity of the isolates was checked by microscopic observation and further confirmed by denaturing gradient gel electrophoresis (DGGE) analysis of PCR-amplified 16S rRNA gene fragments (Teske et al. 1996).

### Phenotypic characterisation

Substrate utilisation by the isolated strains was determined in duplicate in the same bicarbonate-buffered medium as used for enumeration and isolation but without yeast extract. Growth was tested in 100-ml serum bottles closed with butyl rubber stoppers and aluminium crimp seals. The inoculum size was 1% (v/v). The cultures were incubated at 30°C for 5 weeks in the dark. Growth was determined by substrate consumption or product formation as well as by checking for increase in optical density at 660 nm (OD_660_). The following substrates were tested as electron donors in the presence of 10 mM sulfate: 10 mM each of pyruvate, fumarate, butyrate, formate, propanol, ethanol, methanol, serine and cysteine; 5 mM each of malate, glycolate and glycerol; 2.5 mM of benzoate; and 0.5% (w/v) of casamino acids. Sulfite, thiosulfate and 2% (w/v) elemental sulfur were tested as electron acceptors in the presence of lactate as electron donors.

For measuring catalase activity, the fully grown isolates were centrifuged at 13,000 × *g*. The cell pellets were re-suspended on glass slides with a drop of 3% (v/v) H_2_O_2_, bubbles indicated the presence of catalase. Detection of desulfoviridin was performed according to Postgate (1959). Gram staining was performed as previously described (Bartholomew 1962).

### Analytical methods

Acetate and other volatile fatty acids were analysed either by gas chromatography (GC) or high performance liquid chromatography (HPLC). For GC, a Chromopack 9001, equipped with a flame ionisation detector and a fused-silica capillary column 15 × 0.53 mm HP-Innowax, was used. The column temperature was 120°C. The temperature of the injector and detector were 180 and 200°C, respectively. Helium was used as a carrier gas. An Aminex HPX-87H column from Bio-Rad (*T* = 60°C) coupled to a UV and a RI detector was used for HPLC; phosphoric acid (0.05 M) was used as an eluent. Sulfide was measured quantitatively by a colorimetric assay (Cline 1969).

### DNA extraction

Genomic DNA was isolated from the bacterial cultures using the Ultra Clean Soil DNA extraction kit (MOBIO Laboratories, California) according to the manufacturer’s protocol. The quality of the extracted DNA was examined on a 1% (w/v) agarose gel and the amount quantified by absorption spectrophotometry using the Nanodrop ND-1000 TM (NanoDrop Technologies, Delaware). Extracted DNA was stored at −20°C until subsequent use in different PCR reactions.

### PCR amplification and DGGE of 16S rRNA genes

For DGGE analysis, amplification of partial 16S rRNA gene was carried out using the primers 341F-GC and 907R as described by (Schäfer and Muyzer 2001), while primers GM3 and GM4 (Muyzer et al. 1995) were used to amplify the nearly complete 16S rRNA gene for sequencing and subsequent phylogenetic analysis. PCR amplification and DGGE was performed as described previously (Schäfer and Muyzer 2001). The quality of the PCR products was examined on 1% (w/v) agarose gel, and the yield was quantified by absorption spectrophotometry using the Nanodrop ND-1000 TM (NanoDrop Technologies).

### DNA sequencing and phylogenetic analysis

The nearly complete 16S rRNA gene fragments, obtained from the strains L3 and L7, were purified using the Qiaquick Gel Extraction Kit (Qiagen, Hilden, Germany). Purified PCR products were sequenced by the company BaseClear (Leiden, The Netherlands). The DNA sequences of about 1,400 bp were first compared to the sequences deposited in public databases using the NCBI BLAST search tool (http://www.ncbi.nlm.nih.gov/BLAST; McGinnis and Madden 2004). Subsequently, the sequences were imported into the ARB software programme (Ludwig et al. 2004) and aligned using the automatic aligner function. The alignment was further corrected manually, and a phylogenetic tree was constructed using the neighbour-joining algorithm with Felsenstein correction.

### rep-PCR fingerprinting

The genetic diversity of the isolates was analysed by rep-PCR (Versalovic et al. 1994) using the primer GTG5 (5O′-gTggTggTggTggTg-3′). The amplification reaction was performed as previously described (Foti et al. 2006). A 1-kb size marker and 600 ng of the PCR product were loaded onto a 1.5% (w/v) agarose gel containing 0.5 × TAE-buffer (200 mM Tris-acetate, 0.5 mM EDTA, pH 8). The electrophoresis was performed for 14 h in a cold room at a constant voltage of 65 V. The gel was subsequently stained with ethidium bromide (0.5 μg/ml) and photographed under UV illumination using the GelDoc UV Transilluminator (Bio-Rad, Hercules, CA).

### Sodium dodecyl sulfate-polyacrylamide gel electrophoresis analysis

Denaturing sodium dodecyl sulfate-polyacrylamide gel electrophoresis of whole-cell protein (in cell-free extract obtained by sonication) was performed on 12% polyacrylamide gels according to Laemmli (1970). The gels were stained with Coomassie Brilliant Blue and photographed using visible light.

### Design of oligonucleotide probes

Specific oligonucleotide probes for the 16S rRNA of the two strains were designed using the Probe Design tool of the ARB software package (Ludwig et al. 2004). The requirements for designing the probes included high specificity, with no organism outside the intended target group having 100% similarity within the target sequence, and the target sequence being located within the high accessibility region of 16S rRNA molecule as suggested previously (Behrens et al. 2003). The probes were named with a number that indicates the position of the first base in the target sequence (by *Escherichia coli* numbering). The oligonucleotides used for in situ hybridisation are given in Table 1.

**Table 1:** 16S rRNA-targeted oligonucleotide probes used in this study

Probe name^a^	Target organisms	Probe sequence (5′–3′)	Reference
EUB338_I	Most bacteria	GCT GCC TCC CGT AGG AGT	Amann et al. (1990)
EUB338_II	Phylum *Planctomycetes*	GCA GCC ACC CGT AGG TGT	Daims et al. (1999)
EUB338_III	Phylum *Verrucomicrobia*	GCT GCC ACC CGT AGG TGT	Daims et al. (1999)
ARCH915	*Archaea*	GTG CTC CCC CGC CAA TTC	Stahl and Amann (1991)
SRB385	Most Deltaproteobacteria	CGG CGT CGC TGC GTC AGG	Amann et al. (1990)
SRB385Db	Some Deltaproteobacteria	CGG CGT TGC TGC GTC AGG	Rabus et al. (1996)
DSR660	Genus *Desulfobulbus*	GAA TTC CAC TTT CCC CTC TG	Devereux et al. (1992)
DSBA1017	*Desulfobacca* acetoxidans	GTT GCC AGG CAC CCC CAT	Dar et al. (2007)
DSV119	*Desulfovibrio* strain L3	GGC AGA TCA TCC ACG CGT	This study
DSV139	*Desulfovibrio* strain L7	CGC TGT TAT CCC GAT CAC	This study

^a^EUB338 is a combination of EUB338_I, EUB338_II and EUB338_III.

### Whole cell hybridisation

Cells from strains L3 and L7 and from the original reactor were fixed, washed and spotted onto Teflon-coated multi-well microscopic slides as described previously (Dar et al. 2007). Hybridisation was carried out according to the protocol described by Manz et al. (1992) using a formamide concentration of 35% (v/v). Quantification of the hybridised cells was performed as described previously (Neef et al. 1996). The hybridised cells were analysed by two independent observers for determining the fraction of positive signal from each probe relative to the signal visualised with general probes for bacteria (EUB338 I, II and III), SRB (SRB385 and SRB385Db) or with the general DNA stain DAPI (4′, 6′ -diamidino-2-phenylindole). In addition, a general probe specific for members of the domain Archaea (ARCH915) was used. The hybridisation experiments were done in duplicate using different fluorochromes for each probe. Different microscopic fields on each slide were analysed to confirm the results. Hybridisation stringencies of the newly designed probes were determined by performing hybridisations with increasing formamide concentrations as described previously (Manz et al. 1992) using target organism(s) and non-target organism displaying three mismatches within the target region.

### Sequence accession numbers

The nearly complete rRNA gene sequences of strains L3 and L7 have been deposited in GenBank under accession nos. EF055876 and EF055877, respectively.

## Results

### Reactor performance

The amount of ethanol dosed to the reactor was continuous and limited to the amount required for sulfate reduction according to the net stoichiometric reaction for sulfidogenic oxidation of ethanol and its major degradation intermediate, acetate. The equations are as follows:

$$ {\text{2CH}}_{{\text{3}}} {\text{CH}}_{{\text{2}}} {\text{OH}} + {\text{SO}}^{{{\text{2 - }}}}_{{\text{4}}}  \to {\text{2CH}}_{{\text{3}}} {\text{COO}}^{{\text{ - }}}  + {\text{HS}}^{{\text{ - }}}  + {\text{H}}^{{\text{ + }}}  + {\text{2H}}_{{\text{2}}} {\text{0}} $$

$$ {\text{CH}}_{{\text{3}}} {\text{COO}}^{{\text{ - }}}  + {\text{SO}}^{{{\text{2 - }}}}_{{\text{4}}}  \to {\text{2HCO}}^{{\text{ - }}}_{{\text{3}}}  + {\text{HS}}^{{\text{ - }}}  $$

The sulfate removal efficiency at the time of sampling was more than 93%. Less than 1 mg of acetate was estimated in the effluent and no significant biogas (CH_4_) production from the oxidation of organic source was observed at the time of sampling.

### Enumeration and isolation of SRB

The maximum number of culturable SRB observed with lactate or ethanol as substrate in the serial dilution tubes was in the order of 10^8^ cells ml^−1^. The tubes with growth in the highest dilution were used for isolation in agar shake tubes. In total, 26 strains were obtained in pure culture, 17 on lactate and nine on ethanol as substrate. Sub-cultivation of the isolated strains was carried out on lactate. Apart from microscopic observation, the purity of the isolated strains was confirmed by single bands obtained from the DGGE of partial 16S rRNA genes amplified from the strains. The DGGE results of the 26 isolated strains identified two melting types (results not shown).

### Genomic fingerprinting

**Fig. 1:**
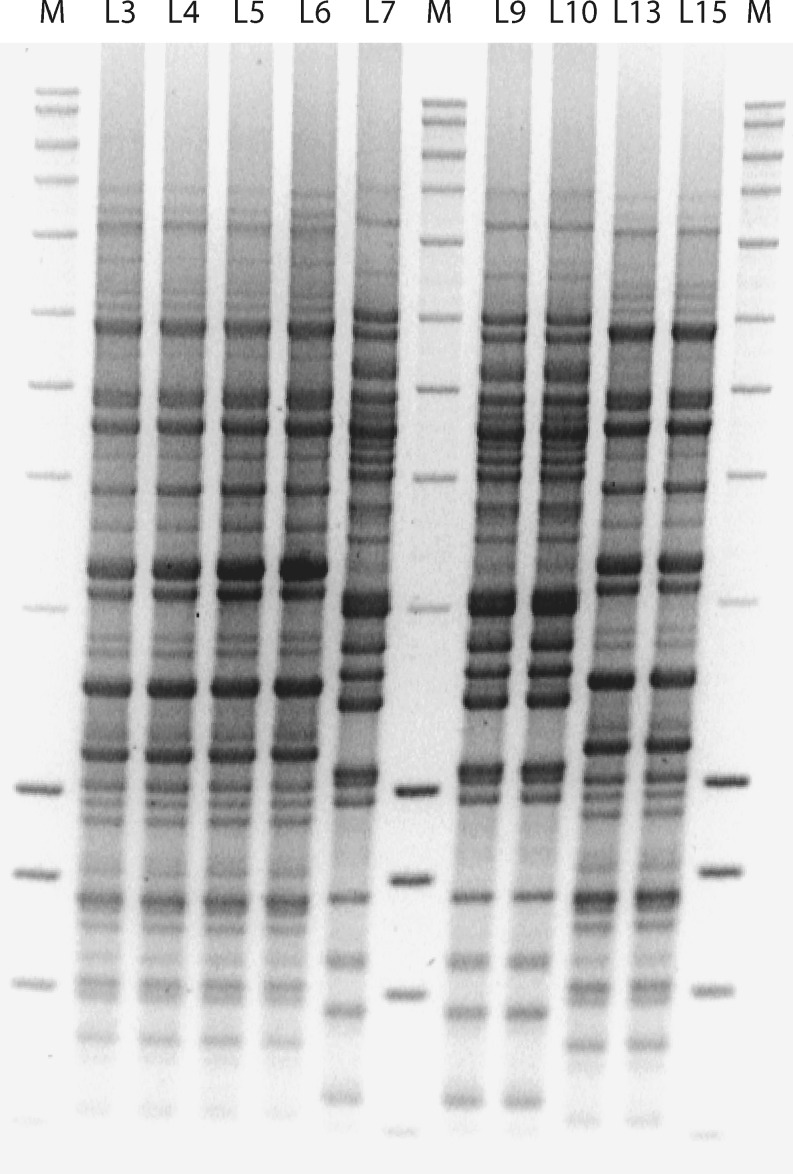
rep-PCR patterns of different strains isolated from the full-scale sulfidogenic bioreactor. *M* is the molecular weight marker

**Fig. 2:**
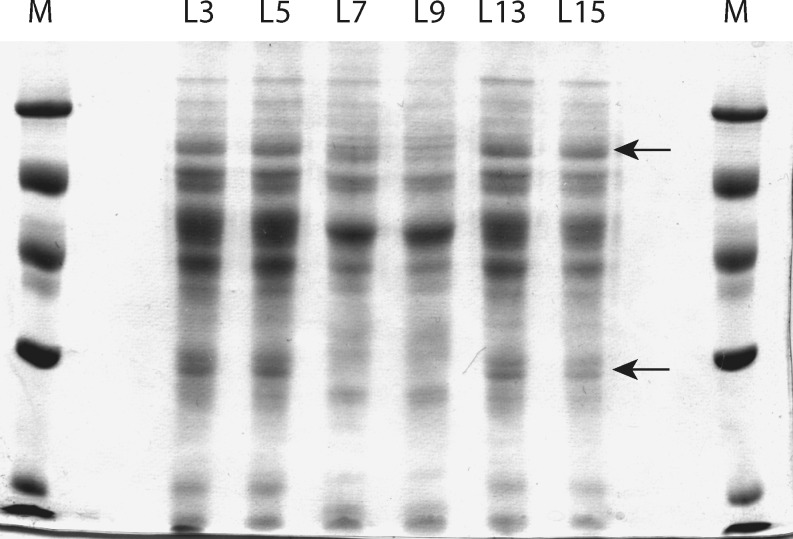
Whole cell protein profile of different strains isolated from the full-scale sulfidogenic bioreactor. *M* is the molecular weight marker. The *arrows* depict protein bands that are expressed in one or the other genotype

The isolated strains were subjected to genomic fingerprinting, i.e. rep-PCR, to resolve any higher degree of genetic diversity among the strains. The rep-PCR profiles of the strains (Fig. 1) revealed the presence of two distinct genotypes with no micro-diversity among the strains. Furthermore, the whole cell protein profile of representative strains of the two groups, grown on the same substrate (i.e. lactate and sulfate) and under similar conditions of pH and temperature, gave only two distinct profiles (Fig. 2), confirming the presence of two genotypes. Two isolates, strain L3 and L7, were chosen as representatives of the two genotypes and were subjected to a more detailed phylogenetic and phenotypic analysis.

### Phylogenetic analysis

**Fig. 3:**
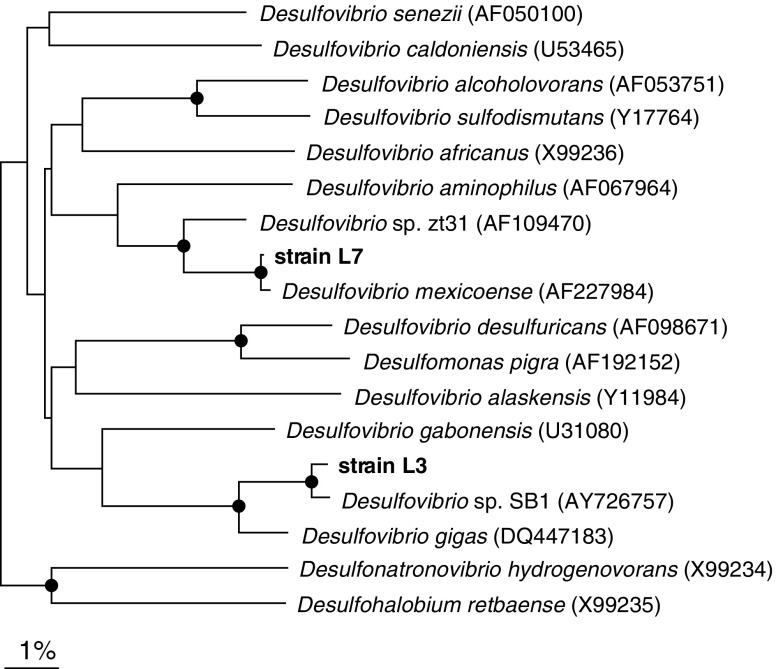
Neighbour-joining tree based on nearly complete 16S rRNA gene sequences showing the phylogenetic affiliation of the two isolated SRB, strain L3 and L7. The sequence of *Desulfobacter postgatei* was used as an outgroup but was pruned from the tree. *Dots* on the nodes indicate bootstrap values of 90% or higher (1,000 replicates).The *bar* indicates 1% sequence difference

A similarity check using the NCBI BLAST search tool of the nearly complete 16S rRNA gene sequences obtained from strain L3 and L7 indicated 98% sequence similarity to *Desulfovibrio* strain SB1 and 99% sequence similarity to *Desulfovibrio mexicoense*, respectively. The phylogenetic affiliation of the obtained sequences is presented in Fig. 3. A neighbour-joining tree based on nearly complete 16S rRNA gene sequences was generated, confirming the close affiliation of the isolated strains L3 and L7 to *Desulfovibrio* strain SB1 and *Desulfovibrio mexicoense*, respectively.

### Phenotypic characterisation of the strains

All isolates had a vibrio to spiral cell morphology. Two distinct groups could be identified based on size and motility. One group of cells related to *Desulfovibrio* strain SB1 was motile, and their size ranged from 4–7 μm in length and 1 μm in width. The other group was non-motile, and the size was 1–2 μm in length and 0.5 μm in width.

Table 2 shows the substrate utilisation patterns of the two isolates. Both strains used lactate, ethanol, pyruvate, glycerol and casamino acids as electron donors. Hydrogen and formate could only be used as substrates in the presence of acetate as carbon source. The organic substrates were incompletely oxidised to acetate. Acetate, propionate, butyrate, glycolate and methanol could not be used as electron donors by both strains. Fumarate and malate were used by strain L3 but not by strain L7, while serine and cysteine were utilised by strain L7 but not by strain L3. Sulfate, thiosulfate and sulfite were used as electron acceptors by both strains. In addition, strain L7 could also use elemental sulfur as an electron acceptor.

**Table 2:** Phenotypic characterisation of strains L3 and L7

Characteristics	Strain L3	Strain L7
Cell morphology	Vibrio	Vibrio
Motility	+	−
Desulfoviridin	+	+
Catalase	+	+
Gram staining	−	−
Electron donors		
H_2_ plus acetate	+	+
Pyruvate	+	+
Lactate	+	+
Acetate	−	−
Propionate	−	−
Fumarate	+	−
Butyrate	−	−
Formate	+	+
Propanol	−	−
Ethanol	+	+
Methanol	−	−
Serine	−	+
Cysteine	−	+
Malate	+	−
Glycolate	−	−
Glycerol	+	+
Benzoate	−	−
Casamino acids	+	+
Electron acceptors		
Thiosulfate	+	+
Sulfite	+	+
Elemental sulfur	−	+

### Whole cell hybridisation

**Fig. 4:**
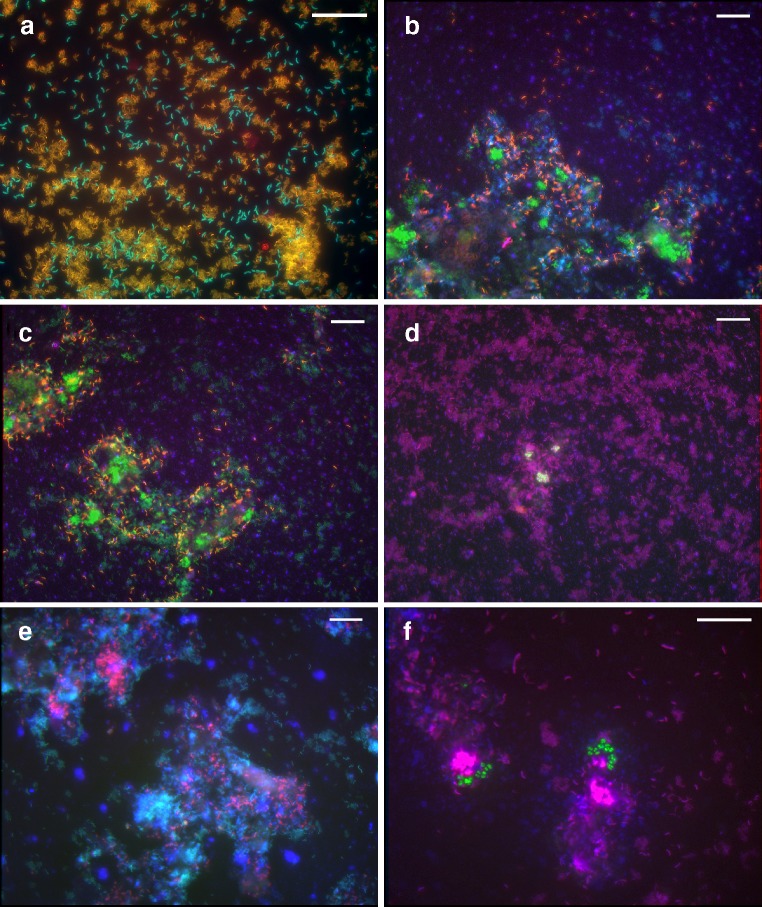
**a** Mixture of cells of strain L3 and L7 hybridised with probe Dsv139 labelled with Cy5 (*blue*), probe Dsv119 labelled with Cy3 (*red*) and probe SRB385 labelled with Fluos (*green*). **b** Sludge sample from a full-scale sulfidogenic bioreactor hybridised with probe Dsv139 labelled with Cy3 (*red*), probe Dsv119 labelled with Cy5 (*blue*) and SRB385 labelled with Fluos (*green*). **c** Sludge sample hybridised with probe Dsv139 labelled with Cy5 (*blue*), probe Dsv119 labelled with Cy3 (*red*) and EUB338 labelled with Fluos (*green*). **d** Sludge sample hybridised with probe DSR660 labelled with Fluos (*green*), probe SRB385 labelled with Cy3 (*red*) and EUB338 labelled with Cy5 (*blue*). **e** Sludge sample hybridised with probe SRB385 labelled with Fluos (*green*), probe DSBA1017 labelled with Cy3 (*red*) and probe EUB338 labelled with Cy5 (*blue*). **f** Sludge sample hybridised with probe Arch915 labelled with Fluos (*green*), probe SRB385 labelled with Cy3 (*red*) and EUB338 labelled with Cy5 (*blue*). Bar is 20μm

After isolation of the most abundant culturable SRB, whole cell hybridisation with specific oligonucleotide probes was performed to estimate the abundance of strains L3 and L7 in the original sludge sample. The specificity of the designed oligonucleotide probes (DSV119 and DSV139) was verified using growing cells of strain L3 and *Desulfovibrio gigas* for probe DSV119 and cells of strain L7 and *Desulfovibrio mexicoense* for probe DSV139. Strain L3 served as a non-target species for L7 and vice versa. A formamide concentration of 35% (v/v) was found stringent enough to discriminate between the two strains (Fig. 4a). In addition, probes specific for cells of *Desulfobulbus* (i.e. DSR660) and *Desulfobacca acetoxidans* (i.e. DSBA1017), which we had detected previously in a similar sulfidogenic reactor (Dar et al. 2007), were also used.

The relative percentage of cells that hybridised with probe DSV119, which is specific for strain L3, was 30–35% of the total SRB385-positive cells and 15–20% of the EUB338-positive cells (Table 3; Fig. 4b,c). The probe specific for strain L7, i.e. DSV139, was detected between 25–35% of the SRB385-positive cell and 10–20% of the EUB338-positive cells (Table 3; Fig. 4b,c). The *Desulfobulbus* and *Desulfobacca*-specific probes also gave a positive signal, but these SRB were somewhat less abundant than *Desulfovibrio* positive cells. The *Desulfobulbus*-specific probe, DSR660, targeted approximately 20–25% of the total SRB cells and 10–15% of the total bacterial cells (Table 3; Fig. 4d). The *Desulfobacca acetoxidans*-specific probe DSBA1017 targeted approximately 15–20% of the total SRB cells and 8–10% of bacterial cells (Table 3; Fig. 4e). The percentage of cells that hybridised with probe specific for Archaea (probe ARCH915) was less than 0.1% of the total DAPI stained cells (Table 3; Fig. 4f).

**Table 3:** Relative abundance of SRB and Archaea^a^

Probe	SRB385	EUB338	DAPI
DSV119	30–35	15–20	12–16
DSV139	25–35	10–20	8–16
DSR660	20–25	10–15	8–12
DSBA1017	15–20	8–10	6–8
ARCH915	–	–	<0.1

^a^Percentage of positive cells relative to those detected by a mixture of probes SRB385 and SRB385Db (SRB385), a mixture of probes EUB338 I, II and III (EUB338) and to cells stained with the DNA stain DAPI.

## Discussion

Based on the observed sulfate removal efficiency (>93%) and the amount of ethanol dosed to the reactor, it may be assumed that a major part of electron flow from the substrate is scavenged by the SRB. The presence of less than a milligram of acetate in the effluent stream with no significant production of CH_4_ points to the oxidation of ethanol mainly through sulfate reduction, although fermentation of ethanol through acetogenesis cannot be ruled out. The metabolic products of fermentation and acetogenesis, mainly acetate and hydrogen, may serve as substrates for methanogens and sulfate reducers; however, under high sulfate concentrations in the reactor, hydrogen and acetate would be more readily used by hydrogenotrophic and acetate-utilising sulfate reducers, respectively, because of more favourable substrate affinity (K_s_) values of the SRB for these substrates (Stams et al. 2005). Fermentation of ethanol with propionate as the major reduced end product has also been described in the literature (Schink 1984; Tholozan et al. 1992); and under such fermentation, propionate will serve as an energy source for the members of the genus *Desulfobulbus* among the SRB.

### Genetic diversity among the dominant culturable isolates

Compared to high micro-diversity among phylogenetically similar strains detected in sediments (Sass et al. 1998; Wieringa et al. 2000; Klepac-Ceraj et al. 2004), no such micro-diversity was observed within the 26 strains isolated from the bioreactor. The chemical complexity of sediments with steep gradients of substrate concentrations, redox potential and pH may give rise to a number of physico-chemical and depth-defined micro-niches, resulting in the evolution of co-existing but genetically distinct sub-populations (Gray et al. 1999). In addition, Torsvik et al. (2002) suggested structural complexity of sediments being an important factor that allows nutritional partitioning, creating numerous niches that, in turn, results in genetic diversification of populations. From the 26 strains isolated, the ones that had identical 16S rRNA gene sequences were also identical by genomic fingerprinting (Fig. 1) and whole cell protein electrophoresis (Fig. 2). Based on the rep- and protein profiles, two genotypes could be identified among the 26 strains isolated. The absence of micro-diversity among the dominant culturable *Desulfovibrio* populations suggests the existence of a few ecological niches in the reactor. The long-term operation of the reactor under stable operational parameters, like constant temperature, pH, salinity etc., combined with the selection pressure because of nutritional limitation (i.e. the use of a single energy source), might be the reason for absence of micro-diversity. In addition, the upward flow of wastewater through the reactor results in frequent mixing, which, in turn, might prevent the creation of micro-habitats that are assumed to be important for the evolution of genetically distinct sub-populations (Torsvik et al. 2002).

Although the viable cell count of SRB observed (10^8^ cell ml^−1^) was comparable to the previous studies (Vester and Ingvorsen 1998; Oude Elferink et al. 1999; Roest et al. 2005; van Houten 2006), it cannot be ruled out that potential biases associated with culture-based enumeration techniques might have underestimated the overall SRB population diversity.

### Phenotypic and phylogenetic characterisation of the dominant culturable isolates

The physiological characteristics (Table 2) of strain L3 and L7 indicated that they are sulfate reducers. The two isolates oxidised lactate and ethanol incompletely to acetate in the presence of sulfate as electron acceptor. Both used H_2_ and formate in the presence of acetate as carbon source. These metabolic traits, in addition to the presence of desulfoviridin and a typical vibrio-shaped morphology, suggested that they are the members of the genus *Desulfovibrio* (Widdel and Bak 1992). *Desulfovibrio* are Gram-negative sulfate reducers, most of which oxidise their substrates incompletely to acetate (Widdel and Bak 1992). Previous studies have demonstrated the dominance of *Desulfovibrio* species in freshwater sediments (Sass et al. 1998), in oil wells (Voordouw et al. 1996) and in several wastewater treatment plants (Santegoeds et al. 1998; Dar et al. 2005). 16S rRNA sequence analysis of the two strains, L3 and L7, confirmed their affiliation to other members of the genus *Desulfovibrio* (Fig. 3),with *Desulfovibrio* strain SB1 and *Desulfovibrio mexicoense* as closest relatives, respectively. *Desulfovibrio* strain SB1, a mesophilic, Gram-negative SRB was isolated from anaerobic sludge of a gas lift reactor-treating sulfate and zinc-rich wastewater (van Houten 2006); while *Desulfovibrio mexicoense* was isolated from a UASB digester-treating wastewater from a cheese-manufacturing factory in Mexico (Hernandez-Eugenio et al. 2000).

### Co-existing SRB populations in the reactor

After isolation of the most abundant culturable SRB, i.e. the motile strain L3 and non-motile strain L7, whole cell hybridisation using fluorescently labelled oligonucleotide probes was performed to search for their specific signals in the fixed sludge sample. Hybridisation results not only confirmed the presence of the two isolates but also gave an estimate of their abundance relative to the total SRB population and to the overall bacterial community present (Table 3; Fig. 4). Fluorescence in situ hybridisation (FISH) results indicated that the two isolates indeed made up a major part of the hydrogenotrophic SRB community present in the reactor. Cells detected by probe Dsv119, specific for the motile strain L3, appeared as single cells or as chains of three to four cells, while cells detected by probe Dsv139, specific for the non-motile strain L7, appeared as individual cells or as loose aggregates.

According to the ecological principle of competitive exclusion, co-existence of physiologically related populations in the same habitat can be understood if they occupy distinct ecological niches (Gause 1934). Although a number of similarities can be drawn between the strains, L3 and L7, from their substrate utilisation profiles (Table 2), the phenotypic differences together with differences in the range of substrates used by the two isolates may allow them to adapt to slightly different niches within the reactor. The two isolates differed in as many as five physiological properties, which are the use of malate, formate, serine and cysteine as energy sources and sulfur as an electron acceptor (Table 2). The use of elemental sulfur in particular by strain L7 will give it a selective advantage over strain L3 in those places in the reactor where elemental sulfur might be available because of the re-circulation fluid from the aerobic reactor; whereas the motility of strain L3 will confer a competitive advantage to it over strain L7. Potential benefits of motility may include increased efficiency of nutrient acquisition and avoidance of toxic substances (An et al. 2006). The presence of *Desulfobulbus*-like SRB besides the members of *Desulfovibrio* in the same habitat could be explained because of the ability of *Desulfobulbus* to use the fermentation product propionate as an energy source as well; furthermore, under limiting sulfate concentrations, *Desulfobulbus* competes more successfully for ethanol than other sulfate reducers by its ability to ferment ethanol (Laanbroek et al. 1982). The simultaneous presence of *Desulfovibrio* and *Desulfobulbus* in wastewater treatment systems has often been reported in the literature (Nanninga and Gottschal 1987; Raskin et al. 1995; Okabe et al. 2003).

Among the complete oxidisers, the probe specific to *Desulfobacca acetoxidans* gave a positive signal, suggesting their dominance among the acetotrophic sulfate reducers. *Desulfobacca acetoxidans*, first isolated from a sulfidogenic bioreactor (Oude Elferink et al. 1999), is a Gram-negative SRB that can utilise acetate as the only source of organic carbon and electron donor. Kaksonen et al. (2004) also found *Desulfobacca acetoxidans*-like SRB in their lab-scale fluidised-bed reactors that were fed with a single electron donor, i.e. lactate or ethanol.

In summary, this study demonstrated the presence of a consortium of four sulfate-reducing populations in the reactor maintained on a single substrate (ethanol). This is in contrast to the findings of Kaksonen et al. (2004) who reported a relatively more diverse consortium of SRB in a fluidised-bed reactor fed with ethanol or lactate as the only energy source, using clone libraries and DGGE as molecular methods for microbial characterisation. The observed difference in the extent of diversity might be due to the difference in the molecular methods employed. The general probes like DSR660, used during FISH analysis, cannot discriminate among the different species, and the probes like DSV119, DSV139 and DSBA1017 target only the specific populations. A likely complete oxidation of ethanol through sulfate reduction might thus be assumed to be taking place through a combined effort of hydrogenotrophic sulfate reducers (i.e. the motile strain L3, the non-motile strain L7 and *Desulfobulbus* spp.) oxidising ethanol incompletely to acetate and *Desulfobacca acetoxidans* oxidising acetate completely to CO_2_. Genetic diversity analysis of the most dominant culturable *Desulfovibrio* populations suggests that long-term stable operation of a reactor with constant operational parameters and frequent mixing might result in the absence of micro-diversity.
